# Selectable Nanopattern Arrays for Nanolithographic Imprint and Etch‐Mask Applications

**DOI:** 10.1002/advs.201500016

**Published:** 2015-05-06

**Authors:** Hyeon‐Ho Jeong, Andrew G. Mark, Tung‐Chun Lee, Kwanghyo Son, Wenwen Chen, Mariana Alarcón‐Correa, Insook Kim, Gisela Schütz, Peer Fischer

**Affiliations:** ^1^ Max Planck Institute for Intelligent SystemsHeisenbergstr. 370569StuttgartGermany; ^2^ Institute for Materials DiscoveryUniversity College LondonKathleen Lonsdale BuildingGower PlaceLondonWC1E 6BTUK; ^3^ Department of Biophysical ChemistryUniversity of Heidelberg INF 25369120HeidelbergGermany; ^4^ Institute for Physical ChemistryUniversity of StuttgartPfaffenwaldring 5570569StuttgartGermany

**Keywords:** block copolymer micelle nanolithography, glancing angle deposition, nanoimprint lithography, nanoparticle lithography, shadow growth physical vapor deposition

## Abstract

**A parallel nanolithographic patterning method** is presented that can be used to obtain arrays of multifunctional nanoparticles. These patterns can simply be converted into a variety of secondary nanopatterns that are useful for nanolithographic imprint, plasmonic, and etch‐mask applications.

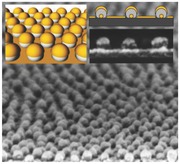

Micro and nanotechnological processes depend critically on lithography and patterning techniques. The development of rapid and precise nanopatterning methods that are time‐ and cost‐effective is a key to fundamental nanotechnology research, as well as a number of industrial processes. For high‐resolution nanopatterns a number of techniques are well developed, including electron‐/ion‐beam‐based litho­graphy^1^, ^2^, ^3^ and tip‐based lithography,^4^, ^5^, ^6^, ^7^, ^8^, ^9^ but they are often too slow for wafer‐scale processes that demand fast processing times. On the other hand, for large‐area nanopatterning, optical/plasmonic lithography,^10^, ^11^, ^12^, ^13^, ^14^, ^15^ contact printing‐based lithography,^16^, ^17^, ^18^, ^19^ and template‐assisted lithography^20^, ^21^, ^22^, ^23^ are promising candidates; however, they require additional expensive and time‐consuming pre‐fabrication processes, such as the preparation of a master template. Colloidal lithography including nanosphere lithography (NSL),^24^ nanoparticle lithography,^25^, ^26^ and block copolymer micelle nanolitho­graphy (BCML)^27^ are attractive large‐scale parallel patterning methods that permit time‐ and cost‐effective patterning at the wafer‐scale. Nevertheless, they suffer from a number of limitations regarding material compatibility, which is an important consideration when preparing etch masks, as well as limitations in the sizes and the shapes of the nanopatterns that can be fabricated by these methods.

Here, we report a nanolithography scheme that combines two established parallel fabrication processes, namely block copolymer micelle nanolithography (BCML) and glancing angle deposition (GLAD). This allows us to grow quasi‐hexagonal nanopatterns of nanodots or other, more complex, nanopatterns on large scales with tunable feature sizes and a wider array of materials than is possible with either method alone. First, this technique extends the sizes of “dots” that can be fabricated to cover the range from BCML (<10 nm) to NSL (>100 nm). This is important for applications in plasmonics for which particle size is closely linked to optical response. We further show that it is straightforward by this method to also change the materials of the nanopatterns and to generate multilayer nanopatterns containing a number of different materials, such as spherical trilayer particles, which prove useful for certain additional processes (including etch masks). By employing wet chemical etching our nanolithography scheme can in addition be used to rapidly fabricate patterns that cannot be obtained with other colloidal lithography techniques, in particular, hollow domes.^24^, ^25^, ^26^, ^27^, ^28^ One important feature of this scheme is that a single multifunctional pattern can, through simple subsequent processing steps, generate a series of novel secondary nanopatterns with different features and 3D morphology. Finally, we present two demonstrations of our nanopatterns in technological applications: we show that they are effective enhancers for surface enhanced Raman spectroscopy (SERS), and we use them as a large area mask for nanoimprint lithography.

BCML is a parallel nanopatterning technique that is used to fabricate highly ordered arrays of spherical Au or Pt nanodots with controllable size and interparticle spacing across an entire wafer.^27^, ^29^ The spatially separated (small) nanodots can serve as nanoscale seeds for the subsequent growth of complex hierarchical nanostructures. While it is possible to enlarge the nanoparticle seeds using electroless deposition,^30^ there is still a limited choice of materials and range of sizes and shapes that can be achieved. To address this, we have chosen physical vapor shadow growth, also known as glancing angle deposition (GLAD), to grow materials onto the nanoseed pattern, as it dramatically extends the range of sizes and materials that can be used as well as the shape of the resulting growth. GLAD is a vacuum deposition technique that can be used to selectively grow nanostructures onto patterns in a parallel manner by exploiting the shadow effect between the constituent seed particles to control the direction of the growth.^31^, ^32^ A key point is that in ordinary (normal incidence) physical vapor deposition the entire wafer would be coated and the nanoseeds would therefore be electrically connected. This is avoided when using shadow growth methods, such as GLAD. Shadow deposition has already been introduced in conjunction with regular patterns formed by e‐beam lithography,^33^ NSL,^34^, ^35^, ^36^ and nanoimprint lithography (NIL).^37^ However, these schemes do not readily permit simultaneous nanometer scale shape control of the patterns and wafer‐scale fabrication. Our previous work combining BCML and GLAD has focused on generating nanoparticles with complex, often chiral, shapes.^31^, ^38^ Using BCML‐grown seeds improved the uniformity of the resulting structures, but the pattern itself has so far been of secondary importance. Here, we concentrate on producing wafer‐level uniform arrays of high symmetry nanodot patterns at below 100 nm for subsequent use, for instance as a simple array of plasmonic structures or as a mask. The symmetry and spacing of the pattern is governed by the BCML step, while the GLAD and following processing steps determine the exact shape of the seed pattern, and hence the polarity (negative or positive) of the mask.

The approach is illustrated by (but not limited to) the three‐step fabrication process depicted in **Figure** **1**. First, a quasihexagonal array of Au nanodots with diameter 12.1 ± 1.8 nm was patterned on a silicon wafer by BCML (Figure 1a). Second, this nanoseed pattern was exposed to a vapor flux in a physical vapor deposition system at an incident angle of 87° with a base pressure of 1 × 10^−6^ mbar to grow hybrid nanoparticles (NPs) upon the nanoseeds using GLAD (Figure 1b). The spacing between the nanoseeds is chosen to be large enough so that the nanostructures do not fuse during the growth process (see the Experimental Section). The deposition process itself consists of three steps: first, we deposited Au on the Au nanoseed pattern to increase the size of the seed particles (if necessary). Next, we deposited Ag as a sacrificial layer. Third, a thin Au film was grown on the array of the Au–Ag hybrid NPs at normal incidence (0°) to cover the entire nanopatterned substrate (Figure 1c). Figure 1d–f shows SEM images of the array of the Au nanodots patterned by BCML (Figure 1d) and the resultant nanopatterned substrate after the three‐step fabrication process. The hybrid NP array can be further processed to create a variety of final patterns, so we call it a multifunctional pattern (see Figure 1e for top view, Figure 1f for tilted view, and Figure S1, Supporting Information, for large‐area SEM images). It can be grown in a very short time (total processing time less than an hour) and consists of an array of Au–Ag–Au hybrid NPs (37.1 ± 4.0 nm in diameter) that cover the entire wafer. We mainly focus on patterns formed by the noble metals Au and Ag since they can be used both as metal etch‐stop layers in a number of different plasma environments^39^ and since they can also function as plasmonically active materials.^40^ However, the fabrication scheme is general and permits the use of a wide selection of materials and material combinations, including alloys.

**Figure 1:**
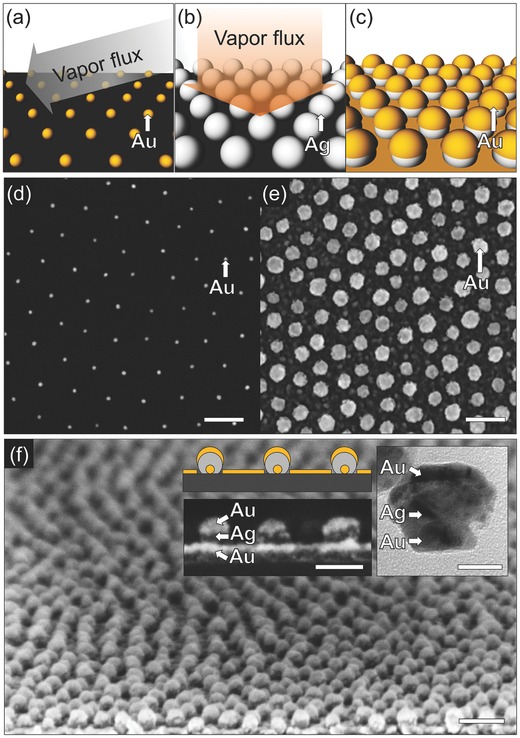
Fabrication of a large‐area multifunctional nanopattern. a) Fabrication of a hexagonal array of Au nanodots on a silicon (or glass) wafer by BCML. b) Growth of Au and Ag on the array of Au nanodots using shadow PVD (GLAD). c) Deposition of an Au thin film on the array of Au–Ag hybrid NPs. SEM images of d) the array of Au nanodots and, e) top view and f) tilted view of the multifunctional nanopattern (scale bar: 100 nm). The inset shows the side view of the SEM image (scale bar: 50 nm) and the TEM image (scale bar: 20 nm) of the multifunctional nanopattern.

A key feature of our scheme is that a series of secondary nanopatterns can be obtained from the initial multifunctional pattern via simple additional processes. In particular, starting from the Au–Ag–Au NP layer, we can generate nanoholes, nanorings, and hollow nanodomes (**Figure** **2**). A nanohole pattern can be obtained by sonicating the multifunctional pattern in water for 1 h (Figure 2c). During the sonication process, the NPs (including the original Au seeds) are removed from the substrate, leaving behind a hexagonal array of circles in the Au film. The spacing of the holes is determined by the spacing of the original BCML array, and their diameter by the size of the Ag in the second growth step. Selective chemical etching of Ag increases the possible secondary nanopatterns that can be fabricated from the multifunctional nanopattern (Figure 2b). Overnight immersion of the multifunctional pattern in a mixture of H_2_O_2_:NH_3_ = 1:1(v:v) produces a pattern of hollow nanodomes surrounding Au NPs with a yield of over 90% (this yield might be enhanced by reducing the Ag etch rate to reduce the rate of bubble generation). The nanocaps (NCs) adhere to the substrate due to significant London–van der Waals attraction between the structures and the substrate with high Hamaker constant of gold of ≈40 × 10^−20^ J in water (Figure 2d).^41^ The diameter of the NCs is again controlled by the Ag deposition step, and the cap thickness by the final Au coverage. Finally, through the sonication process on the hollow nanodome pattern, we can readily lift off the Au NCs from the substrate and this leaves behind the nanohole pattern containing at its center the Au nanodots. We call this pattern a “nanoring” pattern (Figure 2e). The nanodot diameter is controlled by the initial Au deposition that was used to enlarge the original BCML patterned Au nanodots. An important ancillary benefit is that after the sonication processes, the removed NPs are suspended in the supernatant. In the case of the nanohole pattern and the nanoring pattern, sonication yields colloidal Au–Ag–Au hybrid structures and hollow NCs, respectively (see Figure S2, Supporting Information). Thus, this scheme not only generates a patterned surface, but can also be useful in generating colloidal solutions of hybrid NPs. Figure 2f–h shows SEM images of the three secondary patterns. The nanoholes have an inner diameter of 36.7 ± 4.4 nm (Figure 2f), and the nanorings have a gap width of 9.9 ± 1.8 nm separating the central Au NP (21.1 ± 1.5 nm) from the surrounding Au film (Figure 2h). For the nanoring pattern, we have optimized the sonication time to 10 min to yield the nanoring structures with a yield of over 70% of the multifunctional nanopatterned substrate (see Figure S3, Supporting Information). The structural dimensions of such fabricated nanopatterns were in the range of 10–100 nm, which cannot be fabricated by comparable colloidal lithography methods;^24^, ^25^, ^26^, ^27^, ^28^. In particular, the trilayer nanodot, the nanoring, and the 3D hollow nanodome are more complex and smaller than conventional patterns fabricated by GLAD schemes (e.g. dots, rods, rings, triangles, etc.).^33^, ^34^, ^35^, ^36^, ^37^ Additionally, to further illustrate the flexibility of our technique in terms of materials composition, we fabricated a multifunctional pattern with a SiO_2_ top film (instead of the final Au film in Figure 2). Since the SiO_2_ film is CMOS compatible it may be useful for the fabrications of MEMS and CMOS devices, especially 3D devices, based on our patterns.^42^ The resulting nanoring pattern is shown in Figure S5 (Supporting Information). We note that our scheme is capable of growing many different shapes, but of course only shapes that are linear and grow away from the substrate are possible. Shapes that branch, or curve downward toward the substrate can in general not be fabricated, although there are exceptions.^43^ For a more complete discussion of possible shapes, we refer the reader to Ref. 31. Further, the multifunctionality depends on the relative reactivity of the Ag versus Au under attack by H_2_O_2_. Using other materials requires consideration of their reactivities or the choice of solvent. Finally, interparticle spacing defines the size of PVD enlargement that is possible.

**Figure 2:**
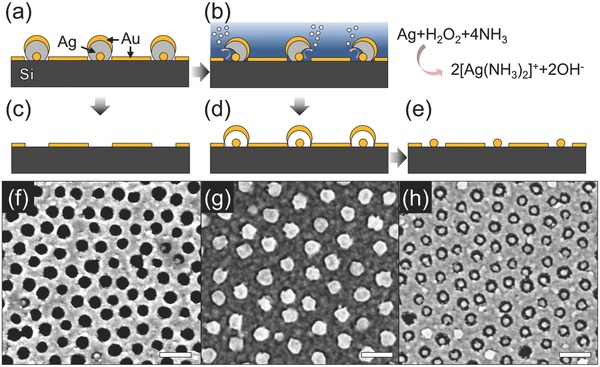
Fabrication of secondary nanopatterns from the multifunctional nanopattern in Figure 1. Schematic views of a) the multifunctional nanopattern as an initial patterned substrate and b) subsequent Ag etching process with the multifunctional nanopattern in the mixture of H_2_O_2_:NH_3_ (v:v = 1:1). The schematic views of the resultant secondary nanopatterns including c) nanohole, d) hollow nanodome, and e) nanoring patterns. The corresponding SEM images of f) nanohole, g) hollow nanodome, and h) nanoring patterns (scale bar: 100 nm).

We believe that our fabrication scheme has a number of potential uses in nanoscience and nanotechnology. Here, we show two applications: patterns for NIL and nanoplasmonic patterns. NIL is useful for large‐scale repeat patterning of entire wafers. Its drawback is the preparation of the initial mask that is expensive and time‐consuming.^17^ We show that our method lends itself to the fabrication of certain NIL master templates (**Figure** **3**a–d). First, a nanohole pattern on silicon substrate was etched using reactive ion etching (RIE) under a SF_6_:O_2_ (3:1) plasma environment, where the etch rate of Si was ≈1 nm s^−1^ (see Figure 3b and Figure S6, Supporting Information). The Au acts as an etch‐stop layer and restricts etching of the Si substrate to the exposed circles within the nanoholes. Next, the Au etch‐stop layer was removed and the patterned Si substrate was covered with a fluoro‐silane for easy detachment of the imprinted surface after the nanoimprint process (see Figure 3c and the Experimental Section for further details). Figure 3d shows an SEM image of the fabricated nanotemplate with ≈120 nm depth. We then tested the templating ability of this master under typical nano­imprint conditions with intermediate polymer stamps (IPS) film (Figure 3e,f). Figure 3g,h shows SEM images of the large‐area arrays of polymer nanowires (NWs) with different heights of ≈30 nm and ≈120 nm by using two different master templates that have different depths (see Figure S7, Supporting Information for a large‐area SEM image). From this, we have confirmed that our patterning technique can be used to create master templates that work well with commercial NIL processes to imprint nanopatterns with soft organic materials. Creating new wafer‐scale template patterns with different feature sizes and periodicity is a simple, parallel, and fast process. We anticipate that these polymer NWs could be potentially useful to produce nanostructured gecko tape^44^, ^45^ or super‐hydrophobic surfaces.^46^

**Figure 3:**
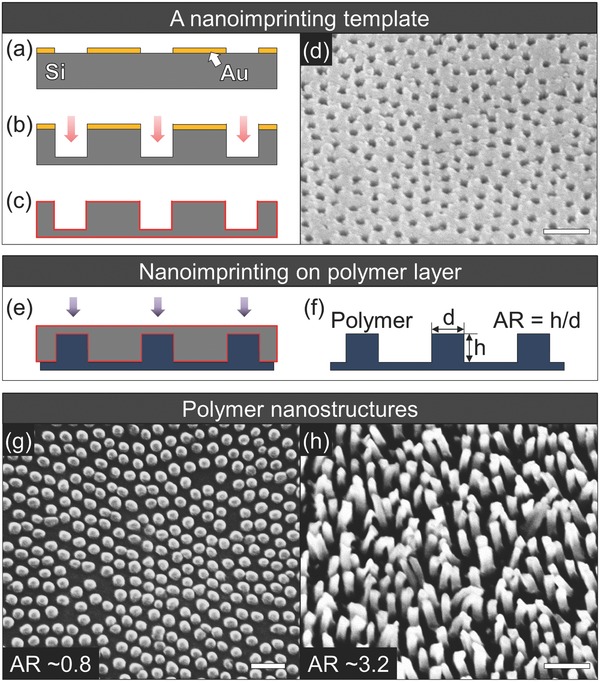
Fabrication of polymer NWs by nanoimprint. a–d) Fabrication of a nanotemplate from the nanohole pattern in Figure 2c. Schematic views of a) the nanohole pattern, b) the patterned substrate after RIE etching under SF_6:_O_2_ plasma environment, and c) the fabricated NIL template after wet‐etching the Au film on the patterns functionalized with a fluoro‐silane. d) SEM image of the fabricated Si nanotemplate with nanohole patterns of ≈40 nm diameter and ≈120 nm depth. e–h) Nanoimprint lithography. Schematic views of e) NIL process and f) the resultant polymer NWs. SEM images of the fabricated polymer NWs with g) AR ≈0.8 and h) AR ≈3.2, respectively (scale bar: 200 nm).

We also investigated the suitability of the grown patterns for plasmonic applications. Measuring the extinction spectra of patterned arrays on quartz glass substrates reveals plasmon resonances that represent the sizes and material composition of the nanomaterials (**Figure** **4**). Normalized UV–vis spectra of the nanoring and the hollow nanodome patterns can be seen in Figure 4a. The extinction of the multi­functional nanopattern is stronger than that of the other patterns and shows a superposition of plasmonic resonances from the Au–Ag–Au NPs and the nanohole patterned substrate. However, in the case of the nanoring and the hollow nanodome patterns, the absence of Ag leads to a sharp dip in the extinction at ≈470 nm that corresponds to the Ag contribution in the multifunctional pattern.^47^ Resonance modes of Au near the wavelengths of ≈610 nm are clearly seen. Moreover, comparison between the spectra from the nanoring and the hollow nanodome patterns shows a peak broadening and blueshift, which we attribute to the contribution from the Au NCs (Figure S2, Supporting Information). The ease with which different patterns with tunable spectral response can be obtained suggests their use for plasmonic‐based sensing,^48^ imaging,^49^ and lithography^12^ applications. Electric‐field enhancement (“hot‐spot” effect) is expected for some of the nanopatterns, for instance at the edge of the nanohole pattern.^50^ Here, the interaction between the Au NCs and the nanohole pattern on the nanoring patterned substrate,^51^ and the nanogaps between the Au NCs and the nanoring patterns on the hollow nanodome patterned substrate,^52^ can be used to obtain defined structures on a large scale. This is in contrast to many nanoparticle structures that either require lengthy electron beam lithography or that are obtained by randomly drying nanoparticles on a substrate.^53^ To test the usefulness of our patterns for surface‐enhanced Raman spectroscopy (SERS), we prepared a hollow nanodome pattern and detected chemisorbed 1,4‐benzenedimethanethiol (BDMT), a common Raman reporter. The SERS signal was measured with 20 s integration time and 0.6 mW power at 633 nm using a He–Ne laser (see the Experimental Section for details). The largest SERS signals were detected on the hollow nanodome patterns at 659.3, 1171.5, 1225.8, and 1593.6 cm^−1^ corresponding, respectively, to the 667.9 cm^−1^ (*ν*_C–S_), 1197.3 cm^−1^ (substituent‐sensitive band of CH_2_ wagging), 1254.3 cm^−1^ (CH_2_ wagging), and the 1607 cm^−1^ (*ν*cc—ring stretch) Raman modes of BDMT.^54^ In contrast to the hollow nano­dome patterns, we found no enhancement of the BDMT signal when we used the ring pattern, which suggests that the enhancement in the case of the hollow nanodomes is indeed due to hotspots in the array structures. In the case of the nanoring pattern, we similarly found SERS effects, probably due to hotspots formed by the 2D gap between the Au seed and its circular hole, and in the case of the nanodomes the 3D gap between the Au seed and the NCs that covers it.^52^, ^55^ We anticipate that the SERS effect can be further increased by optimizing the material composition and feature size of the patterns. This work is currently under investigation by our research group.

**Figure 4:**
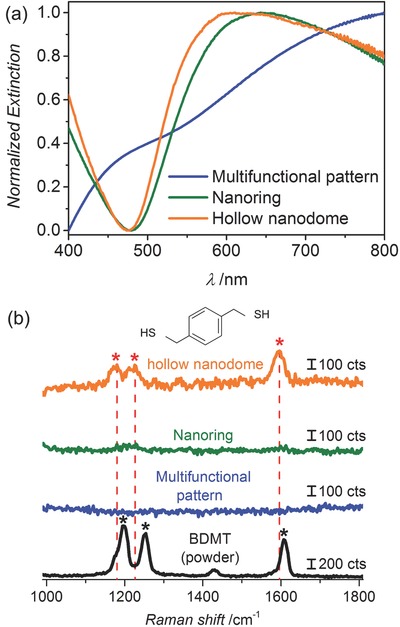
Plasmonic nanopatterns. a) Extinction spectra of the multifunctional nanopattern (blue line), nanoring pattern (green line), and hollow nanodome pattern (orange line). b) Raman spectrum of concentrated BDMT as a reference (black line) and associated SERS spectra from the chemisorbed BDMT on the multifunctional nanopattern (blue line), the nanoring pattern (green line), and the hollow nanodome pattern (orange).

In summary, we describe how the combination of block‐copolymer micelle nanolithography with shadow growth physical vapor deposition, here glancing angle deposition, permits us to turn a pattern of more than a hundred billion well‐spaced nanoseed structures per square centimeter into ones that are plasmonically active with a spectral response that can be shape‐tuned. The method encompasses large areas (entire wafers) that are quickly patterned with regular arrays of nanoparticles as well as tailored 3D structures whose size, material composition, and exact shape can flexibly be tuned. We demonstrate the size‐tuning of hexagonal close packed arrays, as well as the fabrication of wafers with nanoring, nanodome, and nanohole arrays. The flexibility in the use of materials permits their use for plasmonic or nanoimprint applications. The large choice of materials is useful when designing etch masks or for secondary processing to obtain complex nanoparticles. Our approach can readily be extended to fabricate more complex nanopatterns that we expect will be useful for optically, catalytically, electronically, and magnetically active nano‐patterned substrates and devices.

## Experimental Section

*Block Copolymer Micelle Nanolithography (BCML)*: A hexagonal array of Au NPs was fabricated using BCML as a seed layer as previously reported.^27^ Poly(styrene)‐*b*‐poly(2‐vinylpyridine) (S units: 1056; VP units: 671) was dissolved in toluene at a concentration of 4 mg mL^−1^ and stirred overnight. HAuCl_4_•3(H_2_O) was added to the polymer solution at a molar ratio of 0.5 per vinyl pyridine unit and stirred for at least 48 h. This generated self‐assembled spherical micelles loaded at the center with Au salt. To form quasi‐hexagonally close‐packed arrays of the micelles, the micelles were spin‐coated on a 2 in. Si wafer at 8000 rpm for 1 min. After that, the micelles were removed under 10% H_2_ and 90% Ar plasma treatment (power: 350 W, pressure: 0.4 mbar, time: 45 min). In this process, Au was reduced and crystallized as 12.1 ± 1.8 nm NPs with ≈90 nm interparticle spacing.

*Glancing Angle Deposition (GLAD)*: Ag NPs and Au films were grown on the seed layer in a custom‐built GLAD system with independent control over the substrate's azimuthal direction *φ*, and the molecular flux direction *α*. The typical base pressure during deposition was 1 × 10^−6^ mbar. To grow NPs, the flux angle *α* and the azimuthal rotation rates per unit thickness *dφ*/*dθ* were kept to 85° and 12 ± 0.2° nm^−1^, respectively, with closed‐loop feedback based on measurements of material deposition rates using a quartz crystal monitor (QCM). The Au nanoseeds with 12.1 ± 1.8 nm diameter were then expanded by the Au deposition with the tooling factor of 44.5% to 21.1 ± 1.5 nm. Similarly, Ag was grown on the Au nanoseeds with the tooling factor of 12.3% to yield Au–Ag hybrid NPs with a diameter of 37.1 ± 4 nm. The growth of planar films was performed with a 0° tilt of the substrate and without any azimuthal rotation. Figure S1 (Supporting Information) shows the SEM image of the grown multifunctional nanopatterns with thicknesses of the Au films of, respectively, 5, 10, and 15 nm. To increase adhesion between the Si substrate and the Au layer, a 5 nm Cr layer was grown before the Au growth.

*Fabrication of the Nanotemplate*: The nanohole patterned substrate was etched under SF_6_:O_2_ (3:1) plasma environment, where the etch rate of the depth of Si was ≈1 nm s^−1^ (pressure: 8 mTorr, forward bias power: 20 W, ICP power: 100 W, temperature: 20 °C). Figure S6 (Supporting Information) shows the SEM images of the fabricated Si nanotrenches as a function of the etching time and their corresponding structural dimensions. After that, the Au etch‐stop layer was removed by wet chemical etching in the mixture of KI:I_2_ for 1 s and the Cr adhesion layer chemically wet etched in the commercial chromium etchant (Technic Inc.). Finally, the patterned substrate was modified with 1H,1H,2H,2H‐perfluorodecyltrichlorosilane by gas phase silanization for 45 min and followed by incubating in an oven at 85 °C for 1 h.

*Nanoimprint Lithography*: Using thermal and UV nanoimprint lithography (Eitre 3, Obducat), the array of polymer nanowires was fabricated by imprinting the IPS film onto the nanotemplate. The NIL process was performed at a pressure of 40 bar and at a temperature of 160 °C for 60 s. After cooling down to 70 °C, the IPS film was easily peeled off from the template.

*Characterization by SEM and TEM*: The fabricated nanopatterns were imaged by SEM under an accelerating voltage of 5–10 kV. The TEM images of the nanoparticles were recorded on a Philips CM200 TEM under an accelerating voltage of 200 kV. TEM samples were prepared by drop casting ≈10 μL of the colloidal solution onto a holey carbon‐coated TEM grid (Cu 400 mesh), followed by drying under a gentle flow of argon gas.

*UV–Vis Spectroscopy and Raman Spectroscopy*: Extinction spectra of the nanopatterns were measured in the range of 400–800 nm with a resolution of 1 nm on a Cary UV–vis 4000 spectrometer. For the SERS experiments, the nanopatterns were immersed in 1 × 10^−3^ m BDMT dissolved in toluene for 3 h. The samples were then gently washed with toluene and deionized water, and dried. The SERS signals were measured using the 633 nm line of a He–Ne laser for 20 s, where the laser power at the sample was 0.6 mW. The Raman spectrum between 1000 and 1800 cm^−1^ was collected with a spectral resolution of ≈3 cm^−1^.

## Supporting information

As a service to our authors and readers, this journal provides supporting information supplied by the authors. Such materials are peer reviewed and may be re‐organized for online delivery, but are not copy‐edited or typeset. Technical support issues arising from supporting information (other than missing files) should be addressed to the authors.

Supplementary
