# Supplementary material for: Selectable Nanopattern Arrays for Nanolithographic Imprint and Etch‐Mask Applications
Source: Adv Sci (Weinh). 2015 May 6;2(7):1500016. doi: 10.1002/advs.201500016 (PMC5115431; doi:10.1002/advs.201500016)
Supplement: Supplementary file 1 — Supplementary [file ADVS-2-0k-s001.pdf]

Copyright WILEY-VCH Verlag GmbH & Co. KGaA, 69469 Weinheim, Germany, 2015.

ADVANCED SCIENCE
Open Access

Supporting Information

for *Adv. Sci.*, DOI: 10.1002/advs. 201500016

Selectable Nanopattern Arrays for Nanolithographic Imprint and Etch-Mask Applications

*Hyeon-Ho Jeong, Andrew G. Mark, Tung-Chun Lee, Kwanghyo Son, Wenwen Chen, Mariana Alarcón-Correa, Insook Kim, Gisela Schütz, and Peer Fischer**

Supporting Information

Selectable nanopattern arrays for nanolithographic imprint and etch-mask applications

*Hyeon-Ho Jeong[1], Andrew G. Mark[1], Tung-Chun Lee[1,2], Kwanghyo Son[1], Wenwen Chen[1,3], Mariana Alarcón-Correa[1,4], Insook Kim[1,4], Gisela Schütz[1], and Peer Fischer[1,4,]**

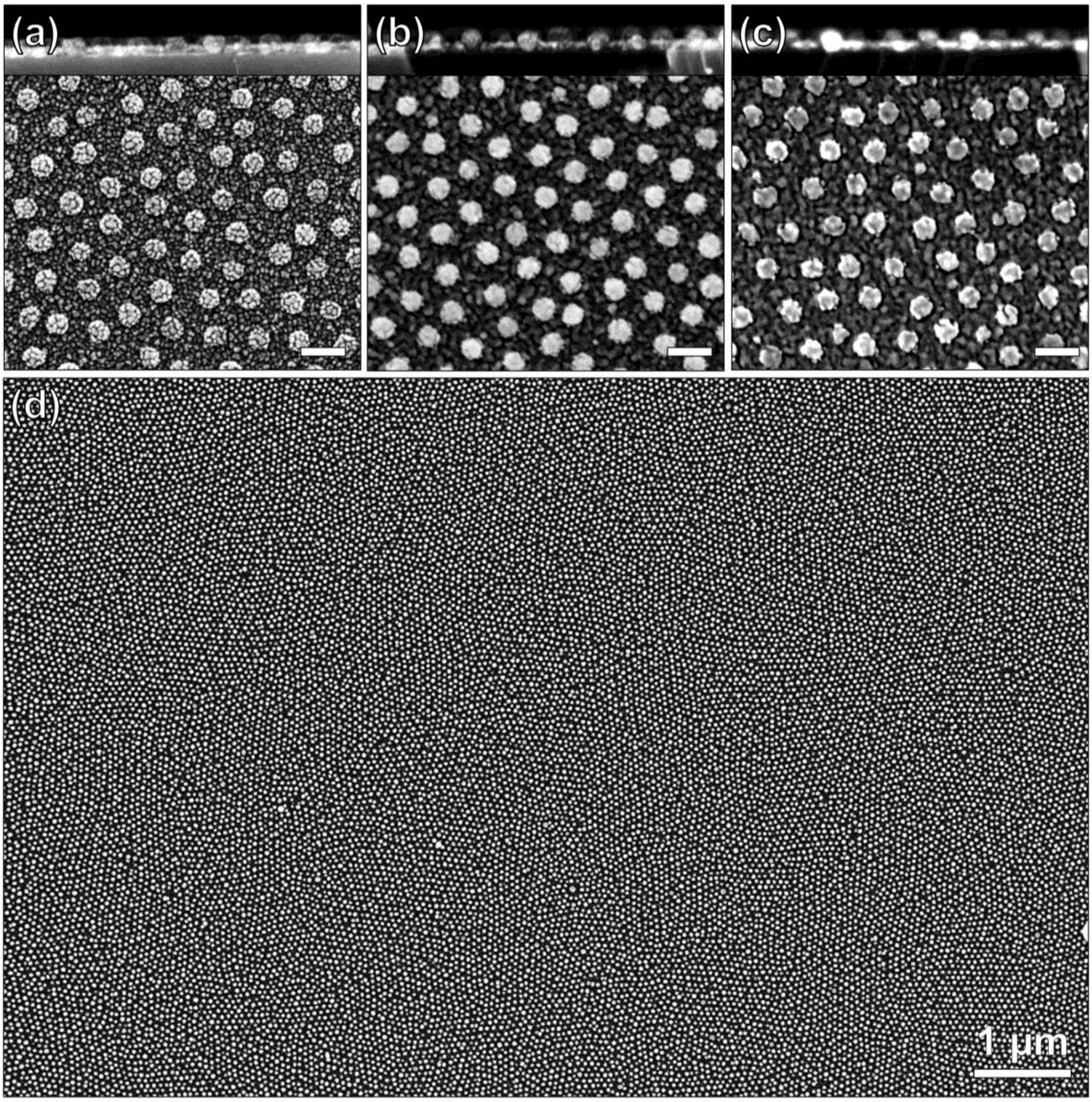

Figure S1. SEM images of the multifunctional nanopatterns with, respectively, a) 5 nm, b) 10 nm, and c) 15 nm thick Au film on the array of Au-Ag-Au NPs (Scale bar: 100 nm). d) Large-area SEM image of the array of the multifunctional pattern with a 10 nm thick Au film.

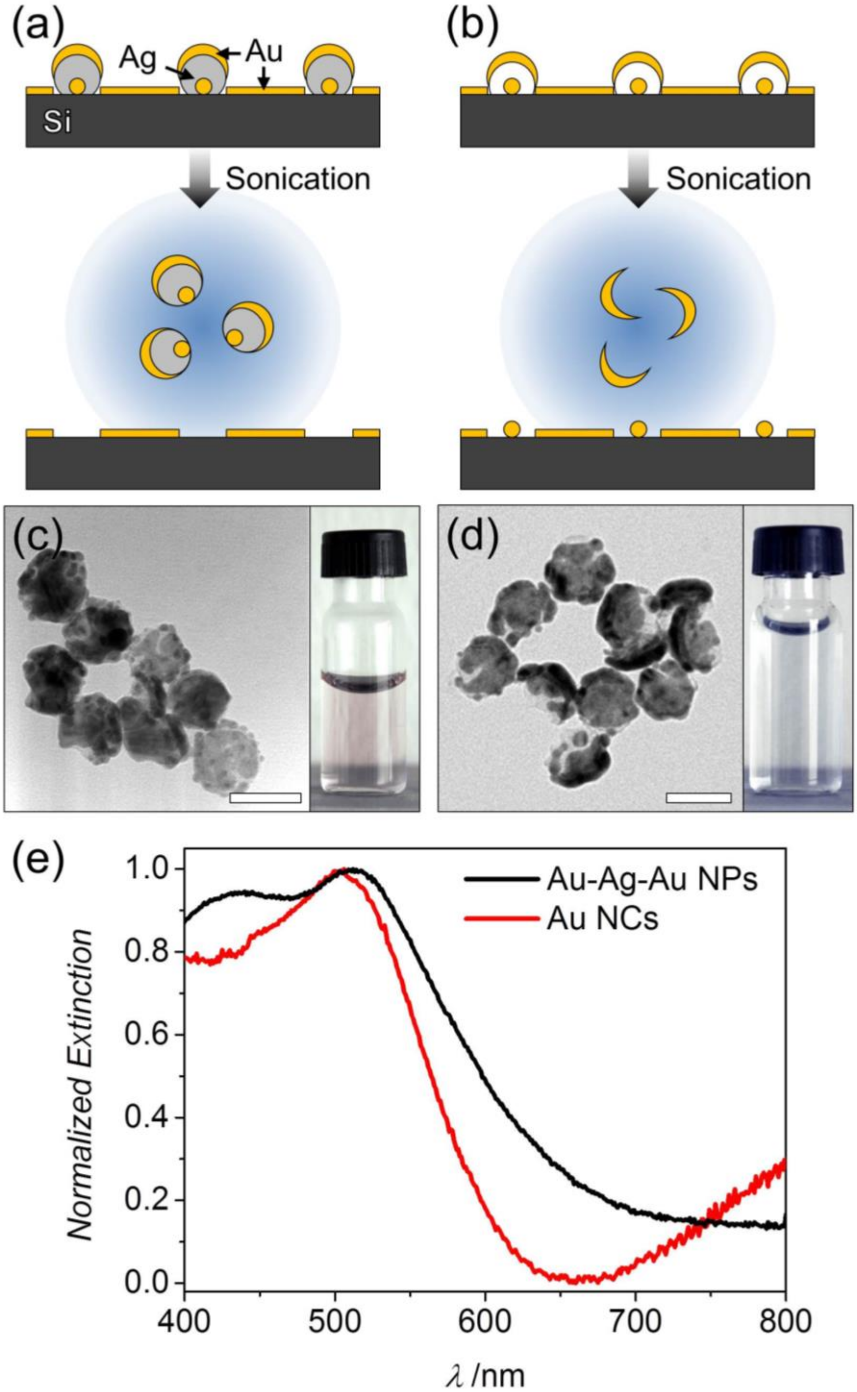

Figure S2. Hybrid nanocolloids. Schematic views of the fabrication of a) colloidal Au-Ag-Au hybrid NPs from the multifunctional nanopattern and b) colloidal Au NCs from the hollow nanodome pattern. TEM images of c) Au-Ag-Au hybrid NPs and d) Au NCs (scale bar: 50 nm). The photos of the 2ml glass vials show the corresponding colloidal solution of the Au-Ag-Au hybrid NPs and the Au NCs. e) Extinction spectra of colloidal Au-Ag-Au hybrid NPs (black line) and colloidal Au NCs (red line).

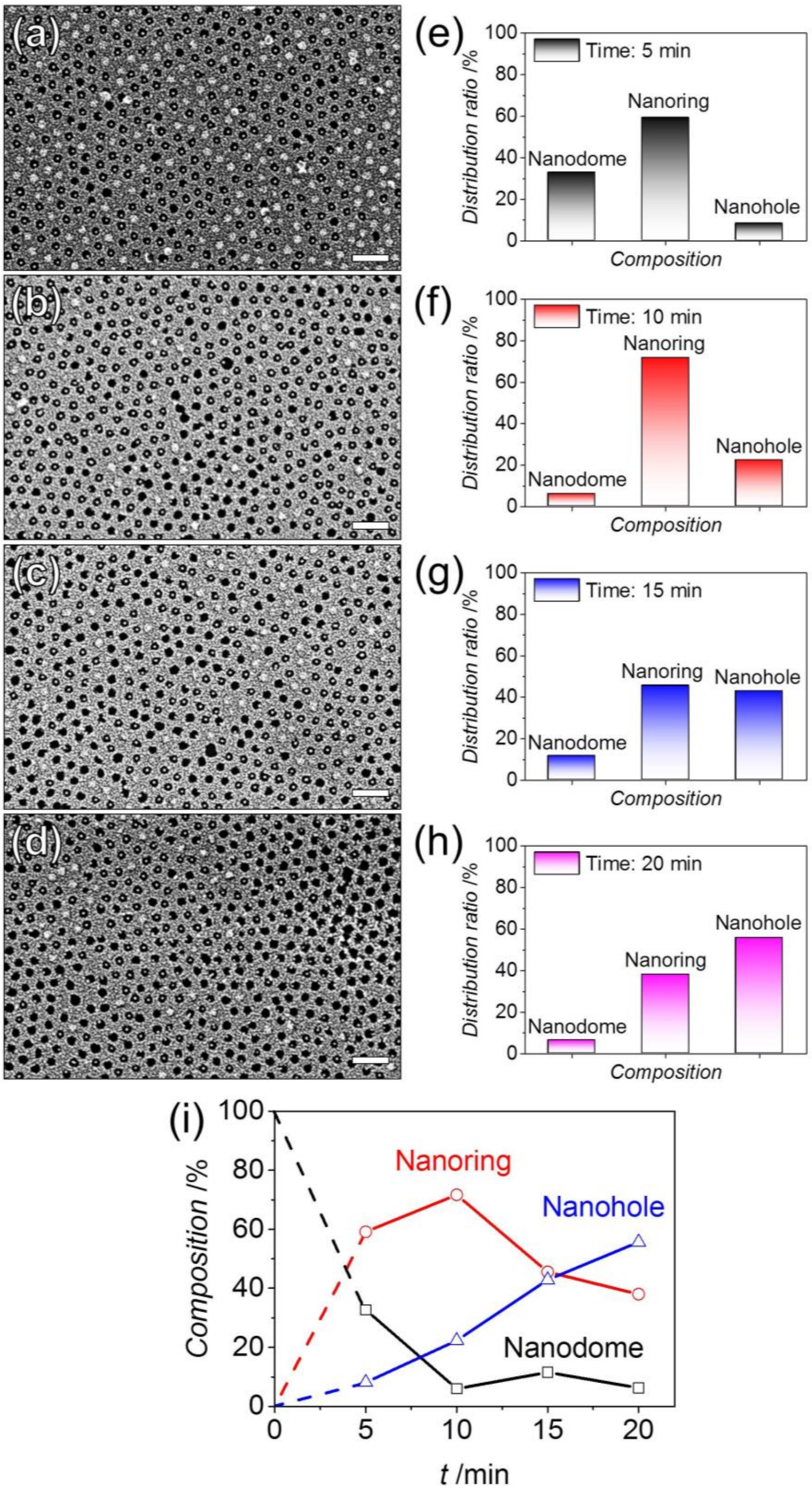

Figure S3. Experimental optimization of the fabrication of the nanoring pattern from the multifunctional pattern by ultrasonication. SEM images of the nanoring patterns after sonication for a) 5 min, b) 10 min, c) 15 min, and d) 20 min, and e)–h) their corresponding distribution ratios for the hollow nanodome pattern, the nanoring pattern, and the nanohole pattern (Scale bar: 200 nm). i) Composition trends of the hollow nanodome pattern (black squares), the nanoring pattern (red rings), and the nanohole pattern (blue triangles) as a function of time.

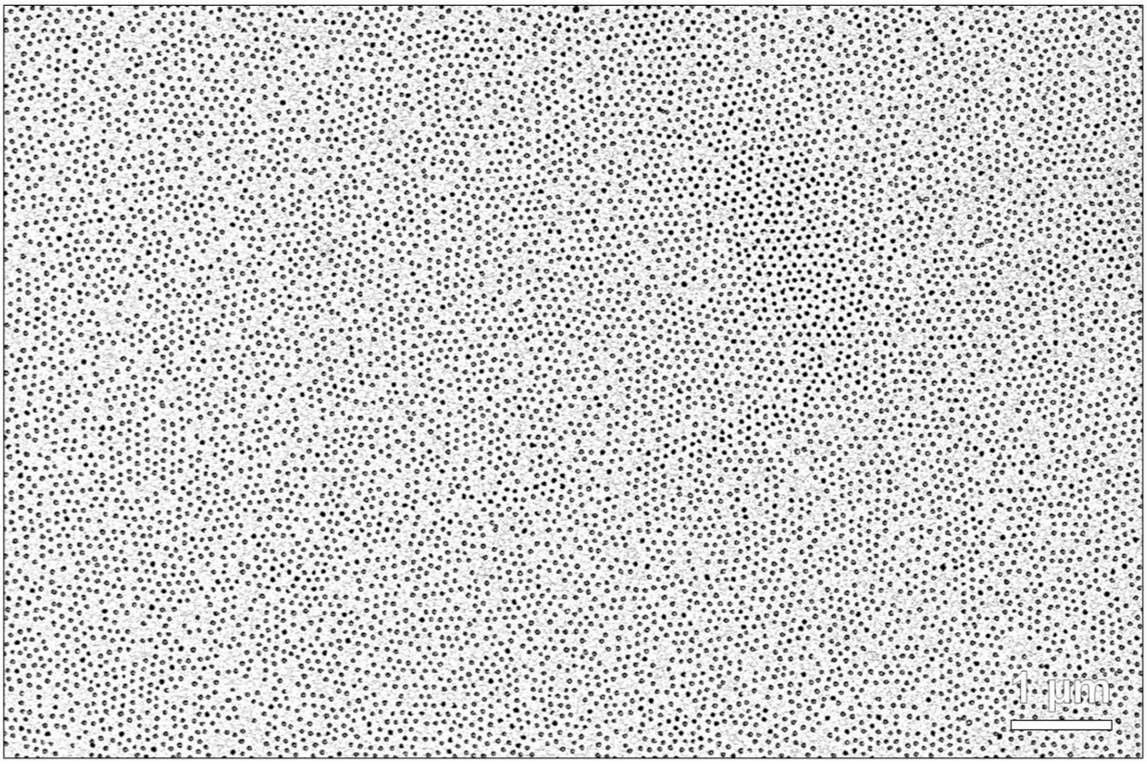

Figure S4. Large-area SEM image of the fabricated nanoring pattern.

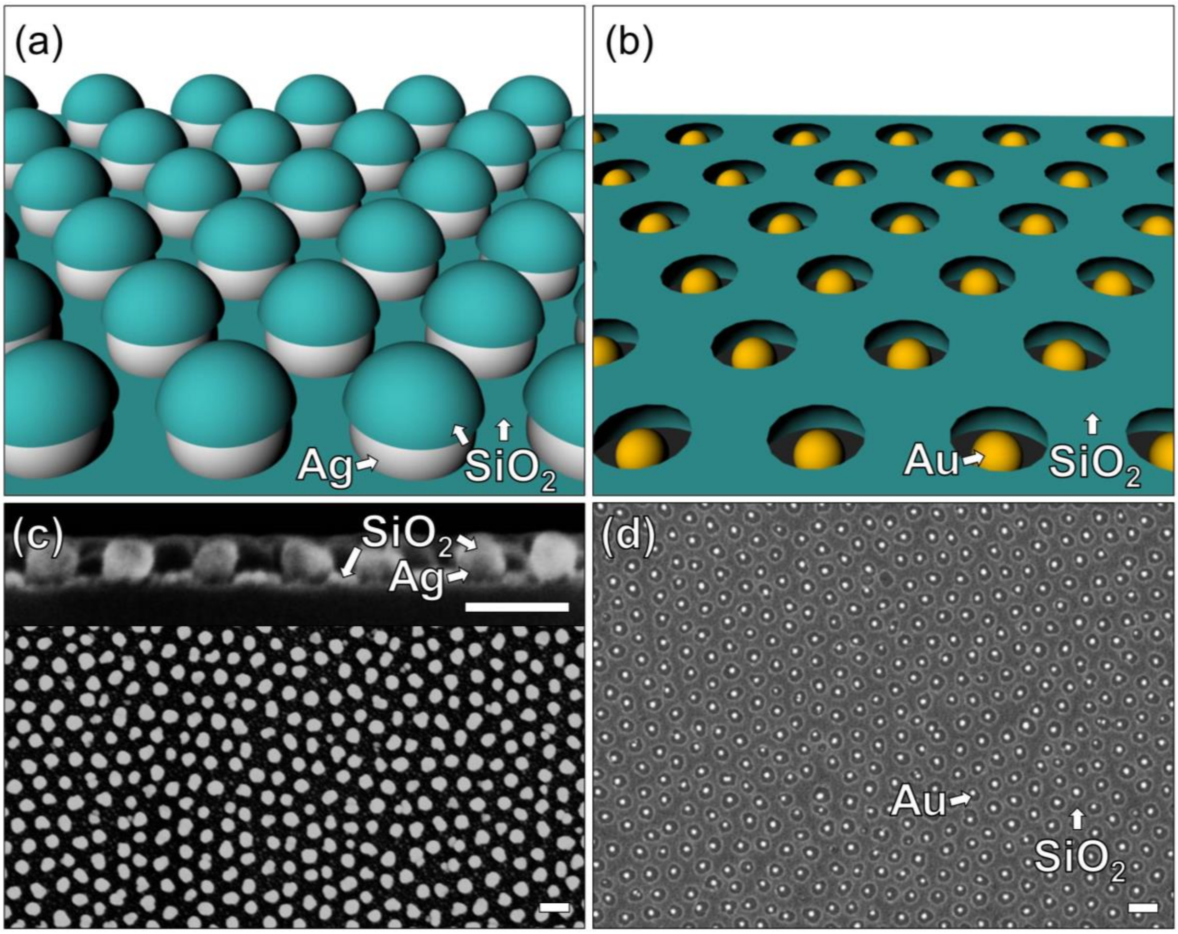

Figure S5. Fabrications of a) multifunctional nanopattern and b) nanoring patterns with a SiO_2 film. SEM images of c) the multifunctional nanopattern (top: side view, bottom: top view) and d) the nanoring pattern (scale bar: 100 nm).

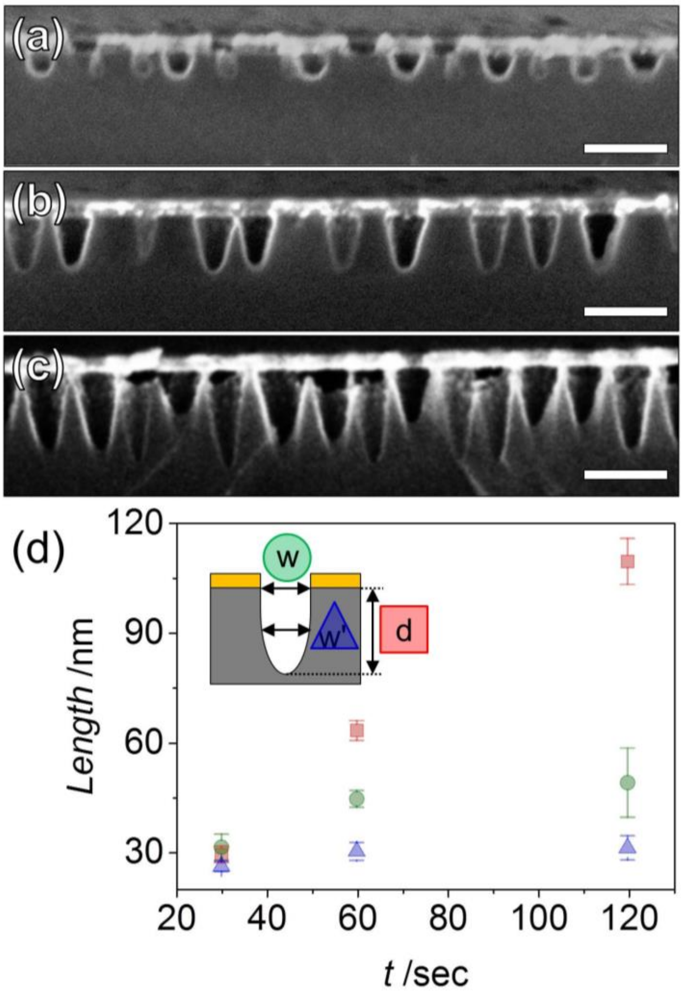

Figure S6. RIE on the nanohole pattern. Side view of the SEM images of the etched silicon substrate after etching for a) 30 s, b) 60 s, and c) 120 s under SF_6/O_2 plasma environment (scale bar: 100 nm). d) The plot of the etched depth (*d*, red square), width (*w*, green circle), and full width at half maximum (*w'*, blue triangle) of Si substrate as a function of time. The etch rate of *d*, *w*, and *w'* were ~0.95 nm/s, ~0.28 nm/s, and ~0.06 nm/s.

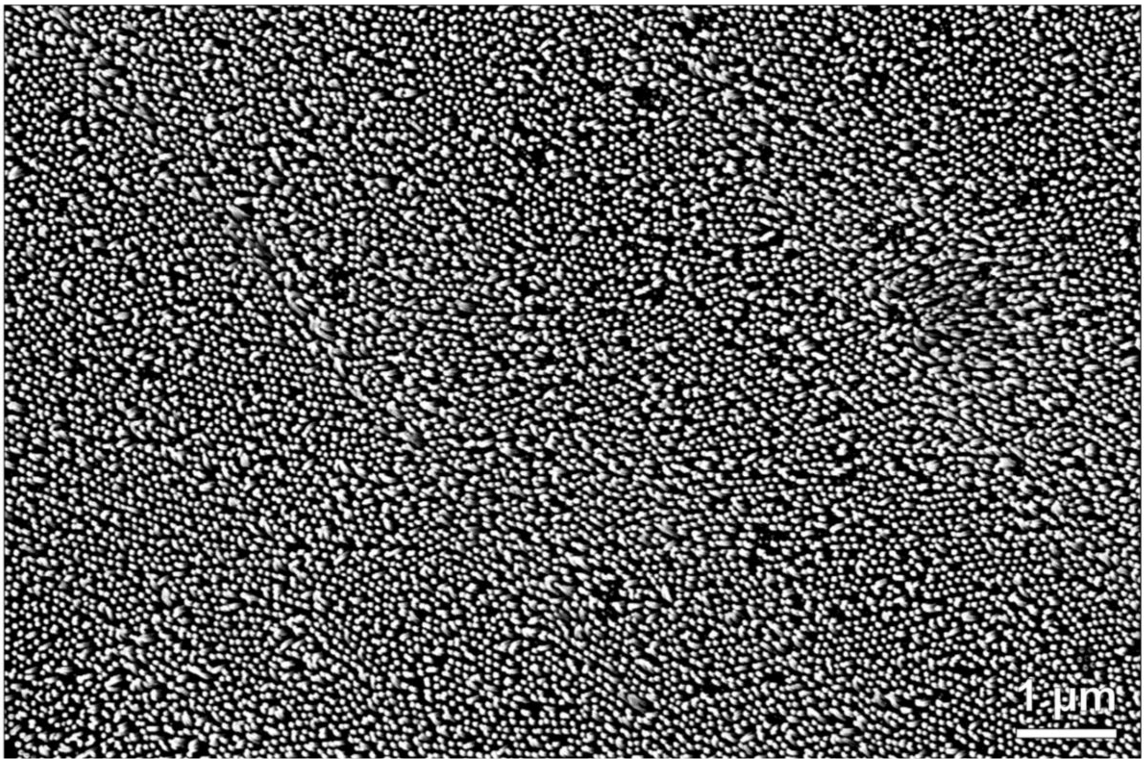

Figure S7. Large-area SEM image of the fabricated polymer NWs.
